# Lineage diversity within a widespread endemic Australian skink to better inform conservation in response to regional‐scale disturbance

**DOI:** 10.1002/ece3.8627

**Published:** 2022-03-14

**Authors:** Duminda S. B. Dissanayake, Clare E. Holleley, Joanna Sumner, Jane Melville, Arthur Georges

**Affiliations:** ^1^ 2234 Institute for Applied Ecology University of Canberra Canberra Australian Capital Territory Australia; ^2^ 2221 Australian National Wildlife Collection CSIRO Canberra Australian Capital Territory Australia; ^3^ 10116 Department of Sciences Museums Victoria Carlton Gardens Victoria Australia

**Keywords:** 2019–20 Australian bushfires, *Bassiana duperreyi*, biogeography, DArTSeq, operational taxonomic units, phylogeography

## Abstract

Much attention is paid in conservation planning to the concept of a species, to ensure comparability across studies and regions when classifying taxa against criteria of endangerment and setting priorities for action. However, various jurisdictions now allow taxonomic ranks below the level of species and nontaxonomic intraspecific divisions to be factored into conservation planning—subspecies, key populations, evolutionarily significant units, or designatable units. Understanding patterns of genetic diversity and its distribution across the landscape is a key component in the identification of species boundaries and determination of substantial geographic structure within species. A total of 12,532 reliable polymorphic SNP loci were generated from 63 populations (286 individuals) covering the distribution of the Australian eastern three‐lined skink, *Bassiana duperreyi*, to assess genetic population structure in the form of diagnosable lineages and their distribution across the landscape, with particular reference to the recent catastrophic bushfires of eastern Australia. Five well‐supported diagnosable operational taxonomic units (OTUs) existed within *B*. *duperreyi*. Low levels of divergence of *B*. *duperreyi* between mainland Australia and Tasmania (no fixed allelic differences) support the notion of episodic exchange of alleles across Bass Strait (*ca* 60 m, 25 Kya) during periods of low sea level during the Upper Pleistocene rather than the much longer period of isolation (1.7 My) indicated by earlier studies using mitochondrial sequence variation. Our study provides foundational work for the detailed taxonomic re‐evaluation of this species complex and the need for biodiversity assessment to include an examination of cryptic species and/or cryptic diversity below the level of species. Such information on lineage diversity within species and its distribution in the context of disturbance at a regional scale can be factored into conservation planning regardless of whether a decision is made to formally diagnose new species taxonomically and nomenclaturally.

## Graphical abstract

Information on lineage diversity within species and its distribution in the context of disturbance at a regional scale is important for conservation planning regardless of whether a decision is made to formally diagnose those lineages as new species.

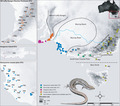

## 1 INTRODUCTION

Species are often regarded as fundamental units of conservation concern and correct species delimitation as essential for an unbiased evaluation of biodiversity in a region or a country (Bickford et al., 2007). As a consequence, much attention is paid to species concepts, to ensure comparability across studies and regions when classifying taxa against criteria of endangerment and setting priorities for action. Lack of comparability lies in part in differing opinions on what should be regarded as species and what should be regarded as lineages within species (Sukumaran & Knowles, 2017). Some authors consider all substantive lineages to be species and that speciation occurs at the point lineages first diverge (Fujita et al., 2012); others follow the biological species concept (Butlin & Stankowski, 2020) and its criterion of reproductive isolation (Mayr, 1942). There are many opinion between these extremes (de Queiroz, 1998), points of view that are often held for operational reasons. In particular, reproductive isolation in allopatry can rarely be definitively demonstrated in context and so presents a fundamental operational challenge to the application of the biological species concept. Regarding lineages as species brings a welcome level of objectivity, and so is favored by some citing operational imperatives, but this approach admits potentially numerous ephemeral entities as species, that is, those lineages that will rapidly admix should the allopatric populations again come into contact (Georges et al., 2018; Kautt et al., 2016; Matute et al., 2020; Momigliano et al., 2017). In this paper, we take the view most recently espoused by Sukumaran and Knowles (2017) that there is a distinction between species in the biological sense and lineage diversity within species.

Were it not for the focus on species‐level taxonomy by legislators, biodiversity assessment could entirely sidestep the issue of species delimitation (Miller et al., 2018). Lineage diversity, whether among or within species, and taking into account phylogenetic weighting (Faith & Baker, 2006), is sufficient as a framework for assessing biodiversity and comparing it through time and across regions. Biodiversity assessment and comparability across geographic and temporal scales has become more prominent as human‐induced impacts extend from the local to regional, continental, and global scales (Pecl et al., 2017). Various jurisdictions now allow entities below the level of species to be factored into conservation planning, as subspecies, key populations, evolutionarily significant units, or designatable units (Green, 2005), and policy development continues in this area (Hoban et al., 2020), but the definitions of these entities are often vague, confusing for managers, and often ignored when it comes to implementation of priorities (Coates et al., 2018; Mouquet et al., 2012; Vane‐Wright et al., 1991).

An excellent case in point of the importance of complete and up‐to‐date taxonomic assessments in conservation planning is that of the regional catastrophic fires in Australia. In the Austral summer of 2019–2020, approximately 97,000 km^2^ of vegetation across southern and eastern Australia was scorched by fires of intensity unprecedented in modern times (Godfree et al., 2021; Ward et al., 2020). Over a billion animals perished in the fires (WWF, 2020), and many species, already endangered, were brought closer to the brink of extinction. But what of cryptic diversity, the diversity represented by lineages within currently accepted species? Species‐level taxonomy lags behind the demands of data used to set priorities for conservation (the “taxonomic impediment”); diversity of lineages within species is arguably even more poorly documented for many species. Yet many of these lineages within species are deeply divergent and some can be regarded as incipient or undescribed species. They are an essential component of biodiversity and their distribution in the context of regional scale disturbance is an important component of conservation planning. Because their ranges are typically narrower than the species to which they are currently assigned, such substantive lineages are particularly vulnerable to regional‐scale catastrophic events, such as widespread bushfires.

Geographical barriers, past paleoclimatic incidents, climatic/environmental factors, and populations surviving glacial maxima in disconnected refugia have played a vital role in the diversification of Australian reptile fauna (Chapple, Chapple, et al., 2011; Chapple, Hoskin, et al., 2011; Dubey & Shine, 2010; Pepper et al., 2018; Rosauer et al., 2015). The family Scincidae represents unsurpassed diversity among vertebrates within the Australian continent (Cogger, 2014; Mitchell et al., 2019). In particular, the forests of eastern Australia are a global biodiversity hotspot and in the top 2.5% of global species richness for lizards (Roll et al., 2017; Williams et al., 2011). Advances in genetic techniques and evolutionary models have accelerated biological studies into isolation and divergence of populations. Current progress in the fields of genomics has opened new opportunities to better understand relationships among the populations within species and their phylogeography.

The eastern three‐lined skink, *Bassiana duperreyi* (Gray, 1838), is a medium‐sized, oviparous scincid lizard, distributed broadly across southern and south‐eastern Australia (Cogger, 2014), including the areas most impacted by the recent fires (Godfree et al., 2021). The widespread nature of this species across a range of bioregions, including cool Alpine, woodland, and heaths to coastal habitat, provides an excellent model to examine lineage diversity within a single species and relate this to habitat alteration and loss. We generated genome‐wide data using the complexity reduction method DArTseq™ (DArT Pty Ltd, Canberra, Australia) which combines next‐generation sequencing to generate a genome‐wide sample of single‐nucleotide polymorphisms (SNPs) (Kilian et al., 2012). This technique has recently become a popular tool for understanding genetic diversity, gene flow, lineage phylogenies, species delimitation, and evolutionary history of a range of organisms for which there is little or no prior genomic information (Georges et al., 2018; Melville et al., 2017).

Our specific goal was to assess lineage diversity within *B*. *duperreyi* in south‐eastern Australia and make an informed decision on which lineages should be regarded as species and which should be regarded as representing substantial diversity within species. We couple our nuclear DNA data with previously published mtDNA data (Dubey & Shine, 2010) to identify substantial lineage diversity within this species of relevance to management, which will provide a foundation for a comprehensive taxonomic evaluation in the future. We also consider the implications for management in the context of widespread fires of lineage diversification within this species.

## 2 MATERIALS AND METHODS

### 2.1 Sampling

**FIGURE 1:**
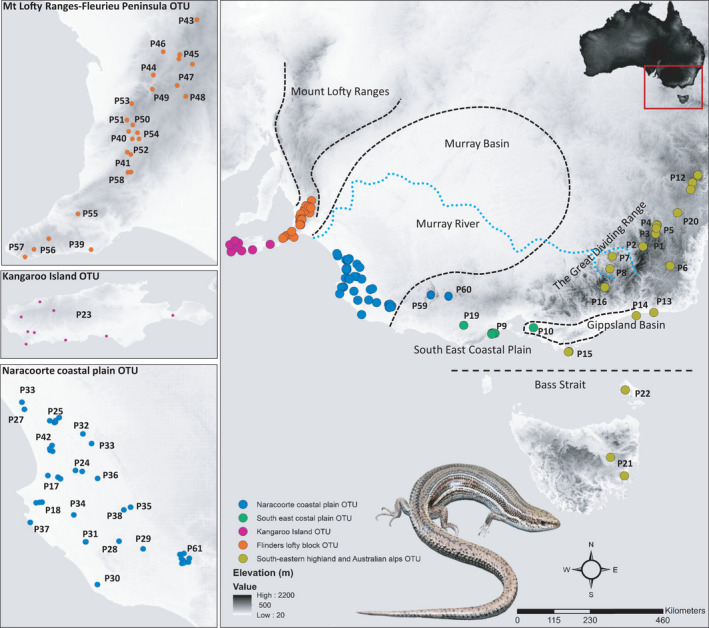
Location of *B*. *duperreyi* populations SNP genotyped in this study from across the range of the species in south‐eastern Australia and including the location of recognized biogeographic barriers. Color scheme is consistent with other figures and OTUs as described in Figure 2. Underlying map generated using ArcGIS 10.5.1 (http://www.esri.com) and data from the Digital Elevation Model (Geoscience Australia) made available under Creative Commons Attribution 3.0 Australia (https://creativecommons.org/licenses/by/3.0/au/legalcode, last accessed 9‐Jul‐20)

We sampled 286 individuals of *B*. *duperreyi* from 63 sample localities across the range of the species, including tissues sourced from museum collections (Museums Victoria and South Australian Museum) and collected in the field (Figure 1, see Supporting Information Table S1). Up to 10 samples per locality were collected when available; for samples obtained from museum collections and field collections, we assigned individuals captured within a 20‐km radius to a single locality. We conducted fieldwork in areas where the lizards were most abundant: in natural open areas, fire trails, or tracks inside the forest areas. Adult *B*. *duperreyi* were captured by hand when actively foraging or under rocks. Tail tips (4–5 mm) were removed with a sterile blade and stored in labeled tubes containing 95% ethanol for subsequent DNA extraction. Lizards were released at their point of capture. All collection protocols were conducted with the permission of Animal Ethics Committees at the University of Canberra and the CSIRO. Description of samples of *B*. *duperreyi* from each locality and their assigned population labels are provided in the Supporting Information Table S1.

Our taxonomic nomenclature follows that of Hutchinson et al. (1990). Outgroup species included were as follows: *Bassiana platynota* (*n* = 2), *Bassiana trilineatus* (*n* = 2), *Pseudemoia pagenstecheri* (*n* = 1), and *Niveoscincus coventryi* (*n* = 1). Nomenclature for bioregions follows that of Australia's bioregions (IBRA) and the description of the recognized biogeographic barriers in eastern Australia, according to Chapple, Hoskin, et al. (2011).

### 2.2 DNA extraction and SNP genotyping

Tissue samples were sent to Diversity Array Technology Pty Ltd. (DArT), Canberra, Australia, for SNP genotyping. DNA was extracted using the NucleoMag^®^ 96 Tissue kit (Macherey‐Nagel, Düren, Germany) coupled with NucleoMag SEP (Ref. 744900) to allow automated separation of high‐quality DNA on a Freedom Evo robotic liquid handler (TECAN, Männedorf, Switzerland). Four combinations of restriction enzymes were evaluated for *B*. *duperreyi* (*Pst*I enzyme combined with either *Hpa*II, *Sph*I, *Nsp*I, and *Mse*I) and the restriction enzyme combination of *Pst*I (recognition sequence 5’‐CTGCA|G‐3’) and *Sph*I (5’‐GCATG|C‐3’) was selected for the complexity reduction by double digestion.

DNA was digested then processed following Kilian et al. (2012), with two different adaptors annealed to the two restriction enzyme overhangs. The library was then subjected to competitive PCR and sequenced using an Illumina Hiseq2500. The sequencing (single‐end) was run for 77 cycles. A full account of the DArTseq™ process used to generate sequences for these samples is given by Georges et al. (2018). The data comprised a matrix of SNP loci by individuals, with the contents stored as 0, homozygote, reference state; 1, heterozygote; and 2, homozygote, alternate state.

### 2.3 Additional SNP filtering

The SNP data and associated locus metadata were read into a genlight object (R Package adegenet; Jombart, 2008) to facilitate processing with package dartR v.1.5.533 (Gruber et al., 2018). Only loci with >99% repeatability (repAvg) were chosen for subsequent analysis. Further filtering was undertaken based on the call rate (>95%), and secondary SNPs where multiple SNPs occurred within a single sequence tag, only one was retained at random. We did not filter for minor allele frequency because this would potentially inflate the counts of fixed allelic differences, particularly given the sample sizes per locality of only *n* ≤ 11. The number of samples per locality was small (*n* ≤ 11), so we could not filter loci for departures from Hardy–Weinberg equilibrium (HWE) or linkage disequilibrium; the sparse sampling of loci across the genome allows the reasonable assumption of little or no linkage between loci. We regard the data remaining after filtering as highly reliable.

### 2.4 Visualization and qualitative analysis

Genetic similarities for individuals and populations were visualized using principal coordinates analysis (PCoA) as implemented in the gl.pcoa and gl.pcoa.plot functions of dartR. A scree plot of eigenvalues (Cattell, 1966), taken in the context of the average percentage variation explained by the original variables (using the diagnostics provided by gl.pcoa function in dartR), guided the number of informative axes to examine.

### 2.5 Genetic diversity

Observed heterozygosity was used as a measure of relative genetic diversity. Heterozygosity was obtained for each population from allele frequencies using the gl.report.heterozygosity function of dartR.

### 2.6 Fixed difference analysis

To examine the possibility that more than one taxon (Operational Taxonomic Unit, OTU) might exist within the geographic distribution of *B*. *duperreyi sensu lato*, a fixed‐difference analysis was done using the scripts gl.fixed.diff and gl.collapse in dartR. An OTU is defined here as an aggregation of populations that can be differentiated by other such aggregations by one or more diagnostic characters. A fixed difference between two populations at a locus occurs when the populations share no alleles at that locus. Accumulation of fixed differences between the two populations is a strong indication of a lack of gene flow. Fixed differences were summed over populations taken pairwise, and when two populations had no fixed differences, they were combined, and the process repeated until there was no further reduction. The resultant OTUs are, by definition, putatively diagnosable at one or more SNP loci. The set of putative OTUs arising from the above fixed‐difference analysis was then tested for significance (using the test = TRUE option in gl.collapse.recursive.r in dartR, (Georges et al., 2018), and pairs for which the number of fixed differences was not statistically significant (number less than expected false positives given the sample sizes) were further amalgamated.

### 2.7 Phylogeny

To evaluate the relationships among individuals of *B*. *duperreyi*, we performed SVDquartets analysis on the individuals grouped by locality (including the individuals from locality: P60 and P59) after further filtration. We selected SVDquartets analysis (Chifman & Kubatko, 2014) because our dataset was composed of short reads with single variable sites per locus (Chou et al., 2015). Heterozygous SNP positions were represented in the dataset by standard ambiguity codes. We used the implementation of SVDquartets in PAUP* v.4.0a165 (Swofford, 2002) with parameters evalQuartets = random, bootstrap = standard, nreps = 10,000 and ambigs = distribute, and designated the *B*. *platynota* and *B*. *trilineatus* as the outgroup. We then assessed the relationships concerning their geographic origin and compared the clades identified with their geographic distributions.

## 3 RESULTS

### 3.1 SNP datasets

The full dataset, which includes all samples, comprised 232,230 polymorphic SNP loci from the sample set comprising samples from 63 sampling localities for the ingroup taxa (n individuals per sampling locality = 1–11, N total individuals = 286) and six outgroup taxa. After stringent filtering on repeatability (repAvg = 0.99) and call rate (0.95), the number of SNP loci in the data set dropped to 66,907 and then 58,906, respectively. A total of 1,655 secondary SNPs, multiple SNPs occurring on a single sequence tag, were also filtered leaving one selected at random (see Materials and Methods). Two outgroup specimens (*P*. *pagenstecheri* and *N*. *coventryi*) and the sole individuals from the Cooma (P6) and Mount Franklin populations (P11) each had an individual call rate of <0.4 (a threshold set taking into account the presence of the outgroups) and were removed from the data. The resultant data, including outgroup specimens (*B*. *platynotus* and *B*. *trilineatus*), are referred to as the full data set, comprising 14,063 polymorphic SNP loci from 60 ingroup sampling locality (*n* = 1–11, *N* = 263) and two sampling localities of the outgroup species (each with *n* = 4). Finally, the *B*. *duperreyi* dataset was obtained by subsetting the ingroup dataset to include only individuals of *B*. *duperreyi* and removing resultant monomorphic loci to yield 12,532 polymorphic SNP loci from 60 sampling localities.

### 3.2 Visualization and qualitative analysis

**FIGURE 2:**
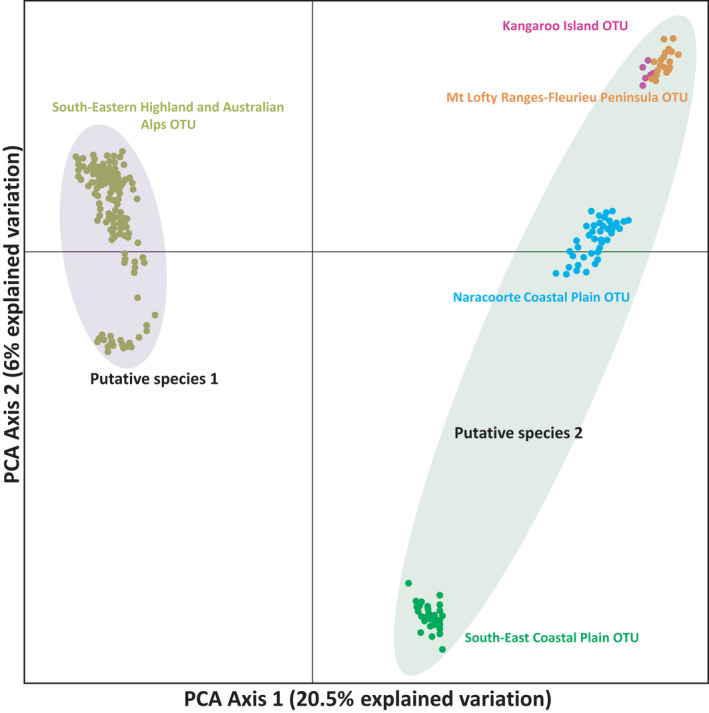
Genetic similarity between individuals using principal coordinates analysis of 12.451 SNP (in group analysis only and recalcitrant individual or population does not present here). Five diagnosable OTUs are defined. Color scheme is consistent with Figures 1 and 3. Refer to text for justification of putative species. Axes not to scale

Preliminary analysis of the data with PCoA applied to the ingroup data set revealed clear evidence of four distinct clusters within the distribution of *B*. *duperreyi* in Australia (Figure 2). Variation represented in Axis 1 (20.5%) separates populations from the south‐eastern highland and Australian alps from populations of the south‐east coastal plain of Victoria and to the west. Axis 2 explains 6% of variation, separating the western block of populations into distinct clusters: Kangaroo Island and Flinders/Mt Lofty Ranges, the Naracoorte coastal plain, and south eastern coastal plain.

### 3.3 Fixed difference analysis

Both of the outgroup taxa *B*. *platynotus* and *B*. *trilineatus*, emerged as diagnosable in the fixed difference analysis applied to the full data set, differing significantly from the expected false positive rate (*p* < .0001). Four individuals Z_60614 (P61), AA80788 (P9), Z_22574 (P60), and Z_22563 (P59) that fell in intermediate positions between major groupings were considered to be examples of contemporary admixture and were removed (following Georges et al., 2018; Unmack et al., 2021). After that, we filtered out 81 monomorphic loci and 12,451 SNPs were retained for further analysis. Five diagnosable OTUs emerged from the fixed difference analysis (Table 1; Figure 2). The 16 populations from the south‐eastern alpine region to Tasmania and including Wilsons Promontory and Flinders Island emerged as the single largest diagnosable OTU (South‐eastern highland and Australian alps OTUs; Figures 1 and 2). Populations from Westernport bay to Corangamite in southern Victoria formed a second diagnosable OTU (South‐east coastal plain OTU), and the genetically distinctive population in the Naracoorte Coastal Plain was defined as a third OTU (Naracoorte Coastal Plain OTU). Populations from the Mt Lofty range extending to southern south Australia formed a fourth diagnosable OTU (Mt Lofty Ranges‐Fleurieu Peninsula OTU), and a genetically distinctive population on Kangaroo Island was identified as the fifth OTU (Kangaroo Island OTU). All OTUs differed by 3–47 fixed differences (See Table 1). These diagnosable OTUs are in broad agreement with the structure evident in the PCoA plot (Figure 2). The fewest fixed differences were observed between Kangaroo Island OTU and Mt Lofty Ranges‐Fleurieu Peninsula OTU, whereas the most were observed between South‐eastern highland and Australian alps OTU and the Kangaroo Island OTU.

**TABLE 1:** Genetic diversity of *Bassiana duperreyi*. Matrix of Euclidean genetic distances (above diagonal) and fixed genetic differences (below diagonal) between the final set of operational taxonomic units to arise from a fixed difference analysis applied to the ingroup data set. Comparisons were based on an average of 12,532 loci after filtering for call rate >95%. All fixed differences were significant at *p* < .0001

	South eastern highland and Australian alps	South east coastal plain	Naracoorte coastal plain	Mt Lofty Ranges‐Fleurieu Peninsula	Kangaroo Island
South eastern highland and Australian alps	0	15.36	16.19	18.83	19.32
South east coastal plain	8	0	13.9	17.05	17.71
Naracoorte coastal plain	7	6	0	12	13.01
Mt Lofty Ranges‐Fleurieu Peninsula	34	5	5	0	8.51
Kangaroo Island	47	37	10	3	0

### 3.4 Phylogenetic inference

**FIGURE 3:**
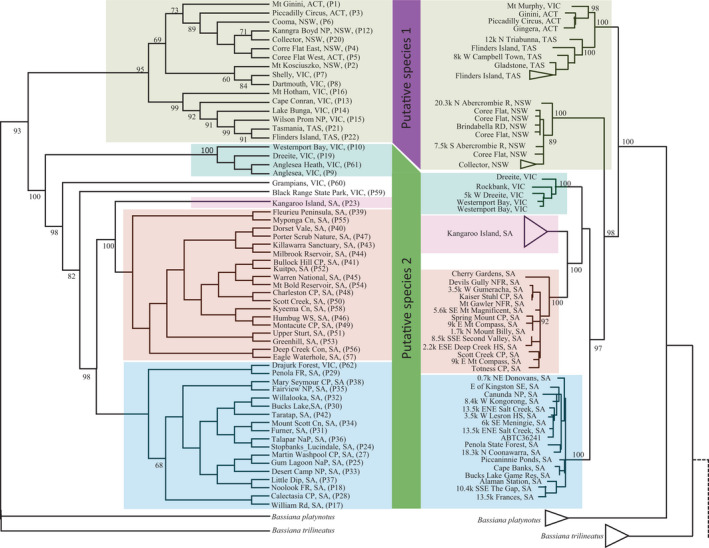
Phylogenetic analyses of Dartseq SNPs with SVDquartets (left) compared to a published phylogeny of two partial mitochondrial genes (ND_2_ and ND_4_) (not to scale) (see Dubey & Shine, 2010). Bootstrap support values are reported for all nodes. Branch lengths are not meaningful for the SVDquartets tree

The fixed difference analysis directs consideration of diagnosability on to the phylogeny to identify those lineages that are diagnostic (Figure 3). The SVDquartets phylogeny of the full phylogeographic dataset had strong bootstrap support across many deeper nodes. Each of the five OTUs identified in the fixed difference analysis emerged as strongly supported clades (> 82% bootstrap support) (Figure 3). The South‐eastern highland and Australian alps OTU showed two distinct sister lineages with 100% bootstrap support. These two lineages include more eastern distribution from the Australian alps (P1 to P8, P12 and P20) and populations from the south east of the distribution, including Mt Hotham (P16), Cape Conran (P13), Wilsons Promontory (P15), Flinders Island (P22), and south east Tasmania (P21). In general, samples were strongly structured according to their biogeographic locations within the Australian bioregion and with associated geographic barriers, with two exceptions: (1) the sample from the Grampians (P60) exhibited substantial divergence from the neighboring Naracoorte populations with 98% bootstrap support; (2) the sample from the Black Range Forest (P59) exhibited a substantial divergence from the Naracoorte Coastal Plain OTU with 82% bootstrap support. There were no significant divergence populations within Naracoorte Coastal Plain OTU. The Kangaroo Island and Mt Lofty Ranges‐Fleurieu Peninsula OTUs showed a 100% divergence between the population, and there were no significant divergence populations recorded within Mt Lofty Ranges‐Fleurieu Peninsula OTU.

## 4 DISCUSSION

This study provides quantification of population structure for the eastern three‐lined skink *B*. *duperreyi* and delineates the distribution of structural elements across the landscape to provide valuable guidance to conservation efforts to protect linage level species diversity in the face of regional scale divergence. Accurate species delimitation of species and structural elements within species are critical for evaluating biodiversity on regional or global scales and for informing effective conservation strategies (Mace, 2004). Failure to appropriately identify species boundaries, structure within species, and the distribution of these across the landscape can have significant consequences for conservation management of imperilled species by diverting finite conservation resources (Agapow et al., 2004). Here, we studied *B*. *duperreyi* as a case study for how within‐species diversity and its distribution across the landscape in the context of regional‐scale disturbance can substantially alter perspectives and priorities set for conservation.

The eastern three‐lined skink *B*. *duperreyi* is currently regarded as a single species (Hutchinson et al., 1990). Mitochondrial variation within this species (Dubey & Shine, 2010) revealed seven geographically localized mitochondrial lineages within *B*. *duperreyi*—referred to as Kangaroo Island, western South Australia, eastern South Australia, southern Victoria, Tasmania, northern Victoria and the Australian Capital Territory, and New South Wales. The genetic distances between the mitochondrial lineages of *B*. *duperreyi* varied from 2.3% (Tasmania cf southern Victoria) to 5.3% (western South Australia cf Tasmania). Given their presumed mitochondrial sequence divergence rate of 1.3% per Myr, Dubey, and Shine (2010) concluded that lizards of the genus *Bassiana* evolved in southern Australia over at least 10 Myr (interspecific divergences), and most of the major intraspecific lineages, including those in *B*. *duperreyi*, diverged between 5.7 and 1.7 Mya. On the basis of these data, they concluded that climatic fluctuations and associated sea level change during the Upper Pleistocene (126,000–12,000 years ago, including the Last Glacial Maximum) did not substantially affect the extent of areas within which the lizards could persist, with the result that the genetic signature of ancient divergence events remains clearly expressed in modern‐day populations as strong geographically associated genetic structure (Dubey & Shine, 2010).

Our nuclear data provide additional evidence of strong geographically associated genetic structure, but with only five well‐supported operational taxonomic units (OTUs) within *B*. *duperreyi*—Kangaroo Island, a Mt Lofty Ranges‐Fleurieu Peninsula in South Australia, Naracoorte coastal plain in southwestern Victoria, the south east coastal plain in Victoria, and an eastern highlands and Australian Alps OTU comprising populations from the ACT, NSW, south‐eastern Victoria, Flinders Island, and Tasmania (Figures 2 and 3). Our five OTUs are broadly consistent with the seven mitochondrial lineages of Dubey and Shine (2010). However, our analysis differs from that of Dubey and Shine (2010) in that they have the Tasmanian, NSW, and ACT lineages (and Mt Murphy, Vic) within a paraphyletic assemblage, outside the remaining lineages of *B*. *duperreyi*, whereas we have the Tasmanian lineage as part of a broader eastern lineage, that is, sister to the remaining lineages to the west (Figure 3). Our largest OTU from the fixed difference analysis combines the 16 populations from the south‐eastern Alpine region (see Table 1) with Tasmania as a single diagnosable OTU, including Wilsons Promontory and Flinders Island. This raises the question as to how this series of populations could have maintained common allelic composition (no significant fixed allelic differences) at all loci over the >1.7 Myr of hypothesized isolation (Dubey & Shine, 2010). A more likely explanation is that the divergence of the mitochondria and the divergence of the lizard lineages do not concur temporally and that there has been episodic exchange of alleles across Bass Strait (*ca* 60 m, 25 Kya, Harris et al., 2003) during periods of low sea level during the Upper Pleistocene. The distinction in mitochondrial sequence between Tasmania and the eastern populations of Victoria, NSW, and the ACT may have arisen through incomplete lineage sorting, preferential dispersal by males or localized selection for what is a highly constrained mitochondrial genome. Hence, the dates of separation of the Tasmanian and mainland populations, estimated to be 1.7 Myr by Dubey and Shine (2010), may apply to the mitochondria but not reflect the history of isolation of the species between Tasmania (incl. Flinders Island) and the mainland, a history that admits exchange during their last terrestrial interconnection in the Upper Pliestocene.

In contrast, both the mitochondrial and nuclear data support isolation of the Kangaroo Island populations from the adjacent mainland that dates back beyond the last terrestrial interconnection between the two (36 m, *ca* 1 Kya). The reasons for this remain speculative, but it is possible that the intervening land bridge between the two areas, when sea levels were lower, contained habitat that was not conducive to the dispersal of *B*. *duperreyi*.

Volcanic activity in western Victoria during the late Pliocene to Holocene and Murray basin (see Schodde & Mason, 1999) could have led to a deep divergence between South‐east coastal plain OTU (Anglesea) and Grampians populations and Naracoorte Coastal Plain OTU of *B*. *duperreyi*. This observation is similar to that made for several other species, including lizards (Ansari et al., 2019; Chapple, Chapple, et al., 2011; Chapple et al., 2005; Ng et al., 2014), amphibians (Schäuble & Moritz, 2001; Symula et al., 2008), marsupial dunnarts (Cooper et al., 2000), and grasshoppers (Kawakami et al., 2009). It is a concordant pattern that is believed to be the result of repeated marine inundation of the area since the Miocene, coupled with the abovementioned volcanic activity. We highly recommend further work on these populations with a larger sample size to understand gene flow between the populations and effect of volcanic isolation. Deep divergence was observed between Kangaroo Island OTU and Mt Lofty Ranges‐Fleurieu Peninsula OTU. The two populations are believed to have split during the Upper Pliocene‐Lower Pleistocene. This agrees with the sea‐level‐driven dispersal opportunities identified by Dubey and Shine (2010).

The level of structure we observed across the range of *B*. *duperreyi* is comparable to other widely distributed species within a geographical range of similar size (Chapple, Hoskin, et al., 2011; Smissen et al., 2013; Symula et al., 2008). This presumably arises as a response to common ecological or geomorphological barriers—such as the Great Dividing Range, the Murray River or habitat structure across the landscape and, as we have discussed, episodic isolation from the mainland of Tasmania, Flinders Island, and Kangaroo Island by rising sea levels. The Murray River appears to be a barrier to dispersal for a range of species (reviewed by Ansari et al., 2019), including lizards, since it extensively dries and breaks into discrete pools nearly once every century (Close, 1990). *Bassiana duperreyi* fits this pattern, and dating studies for this (Dubey & Shine, 2010) and other species suggest a Plio‐Pleistocene diversification in response to barriers afforded by Lake Bungunnia which formed *ca* 2.5 Mya and persisted to *ca* 700 Kya when the modern Murray River was established (McLaren et al., 2011; Stephenson, 1986). It appears that attributes of the modern Murray River and the habitats supported in its basin have been sufficient to maintain signatures of the more ancient divergences in both nuclear and mitochondrial genes of *B*. *duperreyi* (present study) and *Tiliqua rugosa* (Ansari et al., 2019), though the haplotype distribution of the 11 nuclear genes in *T*. *rugosa* were less definitive than the SNP markers were for *B*. *duperreyi*.

Does *B*. *duperreyi* comprise more than one species? We have shown the species to comprise five lineages that have diverged to the point of being diagnosable by one or more corroborated allelic fixed differences. Under some species concepts, diagnosability of a lineage is sufficient to warrant recognition as a species. We take the view, however, that genetic diagnosability, while necessary for a lineage to be considered as a named taxon, is not sufficient (Georges et al., 2018). That is, we admit the possibility of substantial structure within species and take a conservative approach to which diagnosable lineages should be regarded as species as opposed to recognized entities within species (Evolutionarily Significant Units, Management Units, or other Designatable Units). Although there are five diagnosable OTUs, one in particular stands out as particularly distinct, and we have thus identified two putative species within what is currently regarded as *Bassiana duperreyi*. That is, results of both SNPs and the mitochondrial gene tree analysis suggest that the current taxonomy of *B*. *duperreyi* which has it as a single species is not supported and will hopefully prompt revision supported by a combined genetic and morphological analysis. A formal description of the species is beyond the scope of our current study.

Without adequate morphological data, we cannot resolve the taxonomic issues here. However, we propose that there are two putative species within *B*. *duperreyi*. The first is distributed in south‐eastern highland and Australian alpine region (including Wilsons Promontory, Flinders Island, and Tasmanian) and the second is an aggregation of diagnosable lineages (ESUs) occupying the lower elevation regions and coastal regions (including Kangaroo Island, the type locality) (Figure 3). They have broadly parapatric distributions (Figure 1). The south‐eastern highland and alpine taxon is of particular interest, because it has a system of sex determination that involves both differentiated sex chromosomes and sex reversal of the XX genotype to a male phenotype at a frequency that varies predictably with elevation and nest temperatures (Dissanayake et al., 2020, 2021; Dissanayake et al., 2021).

**FIGURE 4:**
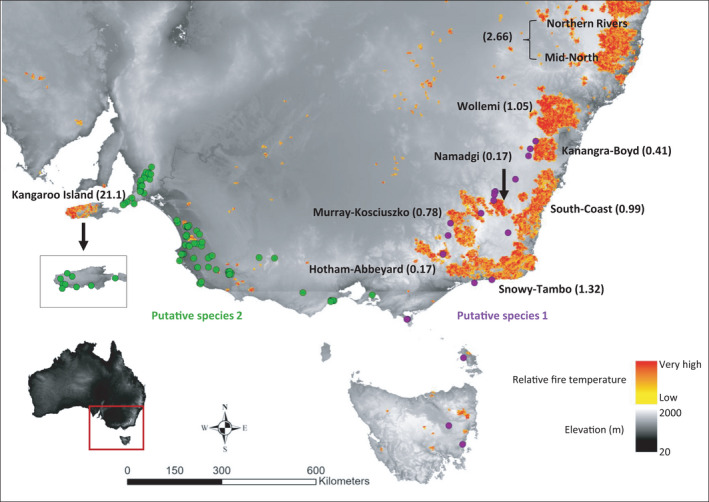
The distribution of *Bassiana duperreyi* in relation to the intensity and extent of the Australian megafire event, which occurred from 1^st^ July 2019 to 11^th^ February 2020. Refer to Supporting Information Table S1 and Figures 1 and 3 for the corresponding population details. The fire intensity and distribution data were obtained from Godfree et al. (2021). The name of the each megafire bracketed value after each megafire is the fire area in millions of hectares. Underlying map generated using ArcGIS 10.5.1 (http://www.esri.com) and data from the Digital Elevation Model (Geoscience Australia) made available under Creative Commons Attribution 3.0 Australia (https://creativecommons.org/licenses/by/3.0/au/legalcode, last accessed 9‐Jul‐20)

Our study has clear management implications in the context of regional catastrophic events and progressive habitat fragmentation and modification at regional levels. The recent fires in Australia were restricted to the eastern mainland portion of the range of *B*. *duperreyi* as currently defined (and Kangaroo Island) (Figure 4). Concern for the impact of the fires is ameliorated somewhat by the existence of substantial populations of *B*. *duperreyi* to the west that were unimpacted by fire. When we consider biodiversity within the species, and in particular the existence of five diagnosable lineages, two of which could be considered putative species, then concerns are reignited. The mainland habitat of the distinctive eastern highlands and Australian Alps OTU (putative species 1 and potentially unnamed) has been severely impacted by fire, which raises concerns for its conservation. Tasmania and Flinders Island provide insurance, but the widespread impact of fire on the habitat of this eastern highlands and Australian Alps lineage is now of serious management concern. Similarly, our demonstration of the distinctive lineage of *B*. *duperreyi* on Kangaroo Island, also seriously impacted by the recent fires, raises its priority for its assessment and management. Our genome‐wide SNP analysis of structure within a widespread species highlights the need for biodiversity assessment at the regional scale to include an examination of cryptic diversity below the level of species and perhaps also a re‐evaluation of species delimitation. Such information on lineage diversity within species and the distribution of those lineages across the landscape can be factored into conservation planning regardless of whether a decision is made to define new species with formal taxonomic decisions.

## CONFLICT OF INTEREST

The authors declare no conflict of interest.

## AUTHOR CONTRIBUTION

**Duminda S. B. Dissanayake:** Conceptualization (equal); Data curation (lead); Formal analysis (lead); Writing – original draft (lead); Writing – review & editing (equal). **Clare E. Holleley:** Conceptualization (equal); Funding acquisition (equal); Methodology (equal); Supervision (equal); Writing – review & editing (equal). **Joanna Sumner:** Methodology (equal); Resources (equal); Writing – review & editing (equal). **Jane Melville:** Methodology (equal); Resources (equal); Writing – review & editing (equal). **Arthur Georges:** Conceptualization (equal); Formal analysis (equal); Funding acquisition (lead); Methodology (equal); Project administration (lead); Resources (equal); Software (lead); Supervision (lead); Writing – original draft (equal); Writing – review & editing (equal).

## Supporting information

Table S1

## Data Availability

All data not available in the paper or supplementary materials are deposited in the Dryad Digital Repository (https://doi.org/10.5061/dryad.tx95x69zc) together with the scripts necessary to replicate the analysis.
